# Kernel Flow: a high channel count scalable time-domain functional near-infrared spectroscopy system

**DOI:** 10.1117/1.JBO.27.7.074710

**Published:** 2022-01-18

**Authors:** Han Y. Ban, Geoffrey M. Barrett, Alex Borisevich, Ashutosh Chaturvedi, Jacob L. Dahle, Hamid Dehghani, Julien Dubois, Ryan M. Field, Viswanath Gopalakrishnan, Andrew Gundran, Michael Henninger, Wilson C. Ho, Howard D. Hughes, Rong Jin, Julian Kates-Harbeck, Thanh Landy, Michael Leggiero, Gabriel Lerner, Zahra M. Aghajan, Michael Moon, Isai Olvera, Sangyong Park, Milin J. Patel, Katherine L. Perdue, Benjamin Siepser, Sebastian Sorgenfrei, Nathan Sun, Victor Szczepanski, Mary Zhang, Zhenye Zhu

**Affiliations:** Kernel, Los Angeles, California, United States

**Keywords:** functional near-infrared spectroscopy, optical brain imaging, tissue optics, time-resolved spectroscopy, optical properties, single-photon detectors

## Abstract

**Significance:**

Time-domain functional near-infrared spectroscopy (TD-fNIRS) has been considered as the gold standard of noninvasive optical brain imaging devices. However, due to the high cost, complexity, and large form factor, it has not been as widely adopted as continuous wave NIRS systems.

**Aim:**

Kernel Flow is a TD-fNIRS system that has been designed to break through these limitations by maintaining the performance of a research grade TD-fNIRS system while integrating all of the components into a small modular device.

**Approach:**

The Kernel Flow modules are built around miniaturized laser drivers, custom integrated circuits, and specialized detectors. The modules can be assembled into a system with dense channel coverage over the entire head.

**Results:**

We show performance similar to benchtop systems with our miniaturized device as characterized by standardized tissue and optical phantom protocols for TD-fNIRS and human neuroscience results.

**Conclusions:**

The miniaturized design of the Kernel Flow system allows for broader applications of TD-fNIRS.

## 1 Introduction

Time-domain functional near-infrared spectroscopy (TD-fNIRS) systems have been considered the gold standard for optical brain imaging systems given their increased information content over continuous wave (CW) systems.^1^^,^^2^ In TD-fNIRS systems, picosecond pulses of light are emitted into tissue, and arrival times of single photons are measured at nearby detectors. The distribution of photon arrival times can be parameterized to estimate tissue optical properties, such as absorption ($\mu_{a}$) and reduced scattering ($\mu_{s}^{\prime }$) coefficients. The photon arrival times can also be used to localize changes in deeper tissues by analyzing the later-arriving photons (“gating”) or analyzing moments of the time of flight (ToF) distribution.^3^

There have been many advancements in TD-fNIRS technology development in recent years that have generated enthusiasm for TD-fNIRS data. Increased numbers of measurement channels,^4^ increased sampling frequencies,^5^ wearable and wireless systems,^6^^,^^7^ and integration of additional data collection methods, such as diffuse correlation spectroscopy,^8^ have improved data quality and enabled broader application areas.

Despite these known advantages of TD- over CW-fNIRS data, widespread adoption of the technology by nonspecialists has been slow due to the paucity of commercial systems.^9^ The few systems that are available commercially have few channels and slow sampling frequencies.^9^ In this paper, we describe our recently developed system, Kernel Flow, which features whole-head coverage and a sampling frequency of 200 Hz for each detector. Kernel Flow was developed for scalable manufacturing, which allows for inexpensive commercial production. We describe the specifications of the Kernel Flow device, benchmark it using the standard basic instrument performance (BIP), nEUROPt, and MEDPHOT protocols,^10^^–^^12^ which leverage tissue and optical phantoms, and show that the system performs well both in the characterization protocols and in localizing brain activation in humans during a standard neuroscience validation task (finger tapping).

## 2 Methods

### 2.1 System Specifications

**Fig. 1:**
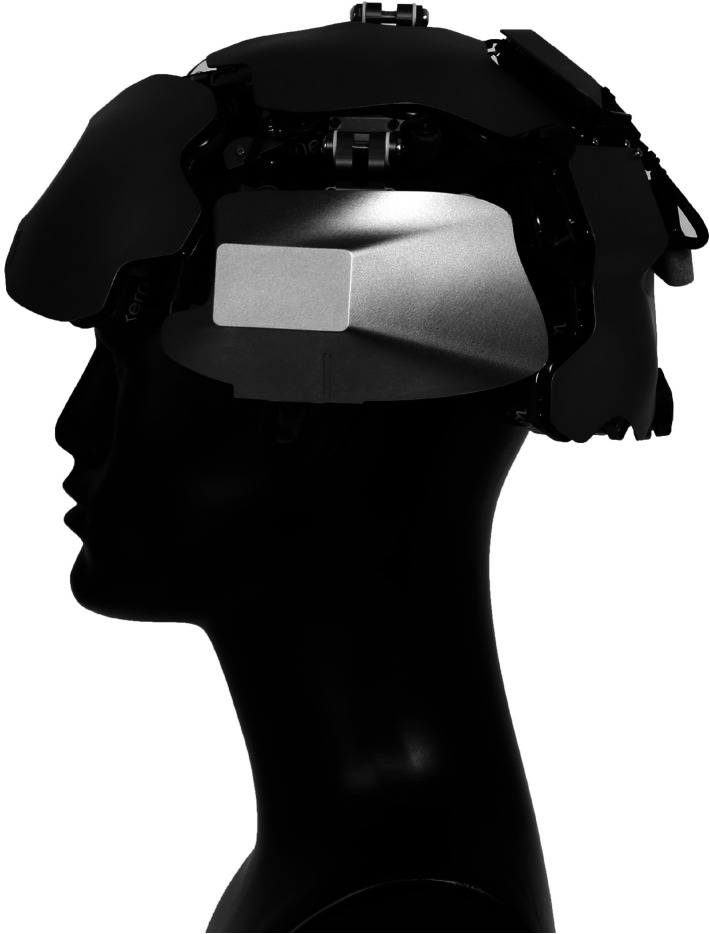
Side view of the modules and structural plates that make up the Flow system. Each plate of modules establishes a fixed distance between the sources and detectors of the modules within the plate. The spacing between plates can be controlled using spacers that should be adjusted based on the user’s head size and desired regions of interest.

The Kernel Flow system consists of 52 modules arranged in a headset design, as shown in Fig. 1. Modules are organized into four plates on each side of the head, covering the frontal, parietal, temporal, and occipital cortices. Each module consists of a central dual wavelength (690 and 850 nm) laser source surrounded by six hexagonally arranged detectors, each 10 mm from the source. Light is transmitted from source or to detector locations using spring-loaded light pipes. The source-detector separation (SDS) within a module is 10 mm, and cross-module channels can also be analyzed. The overall size of the headset is adjustable to fit adult head sizes, with a minimum size of 52.5-cm circumference and a 31-cm bitragion coronal arc. The system weighs 2.05 kg. By comparison, a single detector channel wearable system was reported to weigh 2.5 kg,^7^ although that system includes the weight of the battery while the Kernel Flow system is powered over universal serial bus type-C (USB-C).

**Fig. 2:**
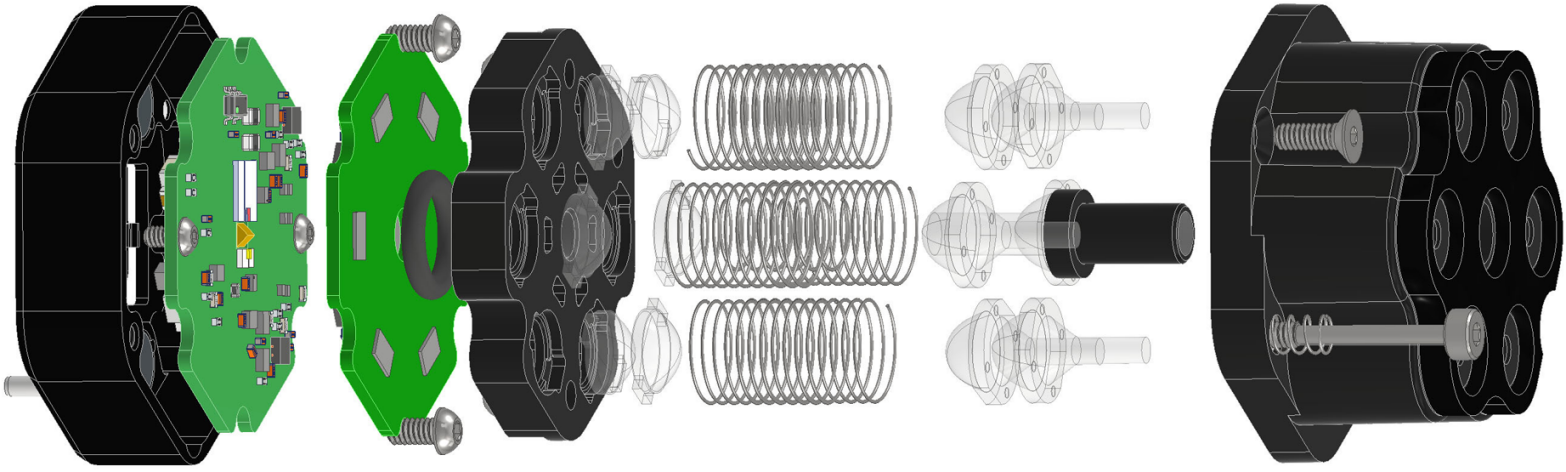
An exploded view showing the details of all of the module subassemblies.

Each module in the system consists of three major subassemblies: laser assembly, detector assembly, and the optical assembly. All three of these subassemblies are shown together in an exploded view in Fig. 2. The details of each subassembly and the overall system architecture are presented in the following sections.

#### 2.1.1 System architecture

**Fig. 3:**
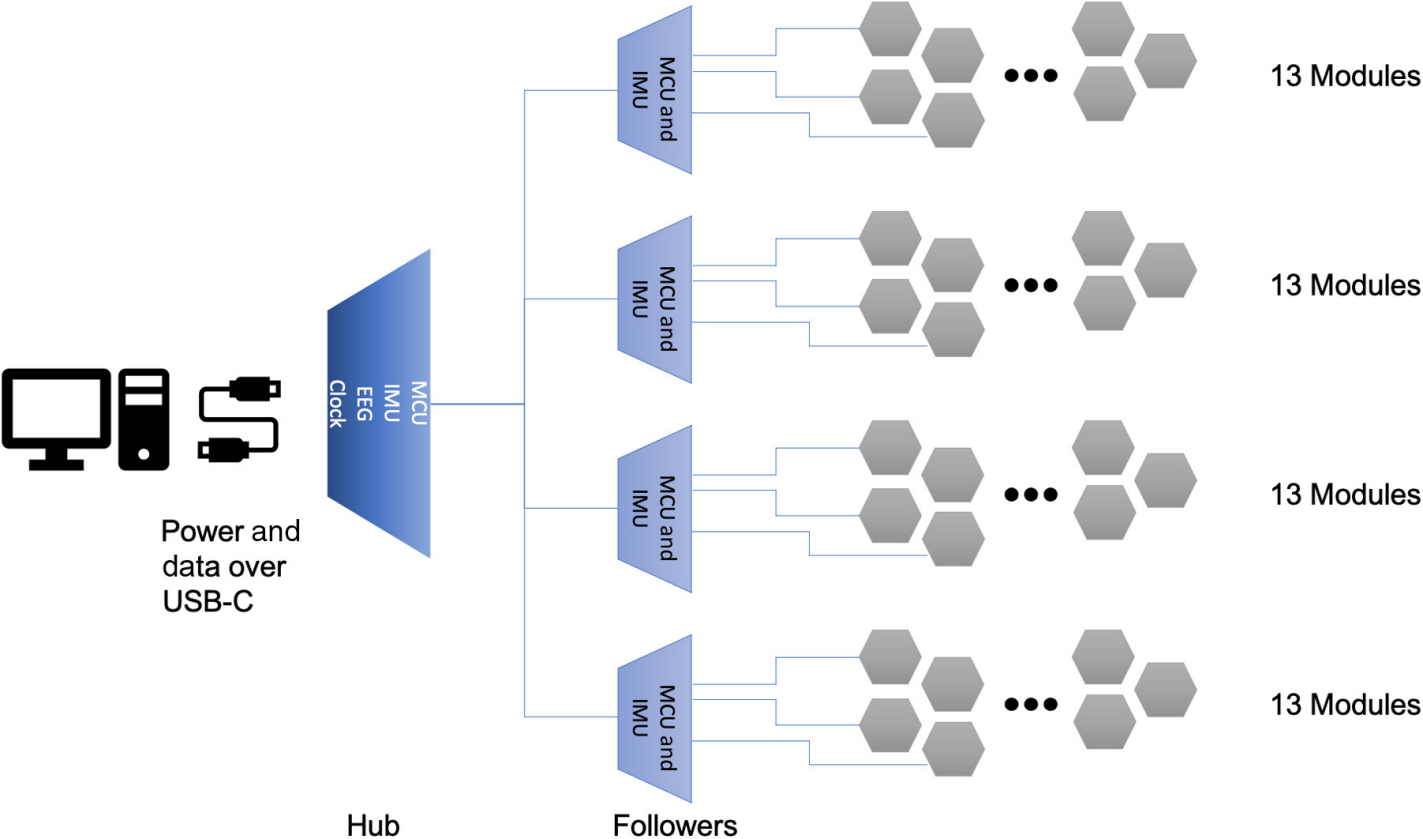
Overall architecture of the Flow hardware. A global clock is distributed from the primary MCU subassembly to all module endpoints.

The Flow system has a hierarchical architecture with electronics and wiring harnesses integrated into the headset, as shown in Fig. 3. The system is cabled over a single USB-C interface that both supplies power and enables bidirectional communication between the data collection computer and the Flow system. The USB-C cable connects to the Flow system through the hub subassembly that includes a microcontroller unit (MCU), nine-axis inertial measurement unit (IMU), and global reference clock. In addition, the hub subassembly includes an eight-channel electroencephalography (EEG) amplifier and analog-to-digital converter that is designed for connecting to active dry electrodes. The hub also handles the primary power negotiation for the USB power delivery standard and distribution of power to the rest of the system. Connected to the hub are four follower boards that serve as data aggregation points for clusters of 13 modules each. Each of these follower boards includes an MCU, additional power conditioning circuitry, and a nine-axis IMU.

In total, the Flow system supports connection of up to 52 time-domain optical modules and includes five nine-axis IMUs, six EEG channels, and self-contained power management and distribution. The system works seamlessly with fewer than 52 optical modules, which enables the removal of any optical modules that are not needed for the intended experimentation. In the human studies reported in this work, we have chosen to populate the entire headset to clearly show regions of activation/deactivation throughout the whole brain during the task. This provides higher confidence in the measured results because artifacts due to systemic physiology and/or motion would be common to all modules.

#### 2.1.2 Laser source module

**Fig. 4:**
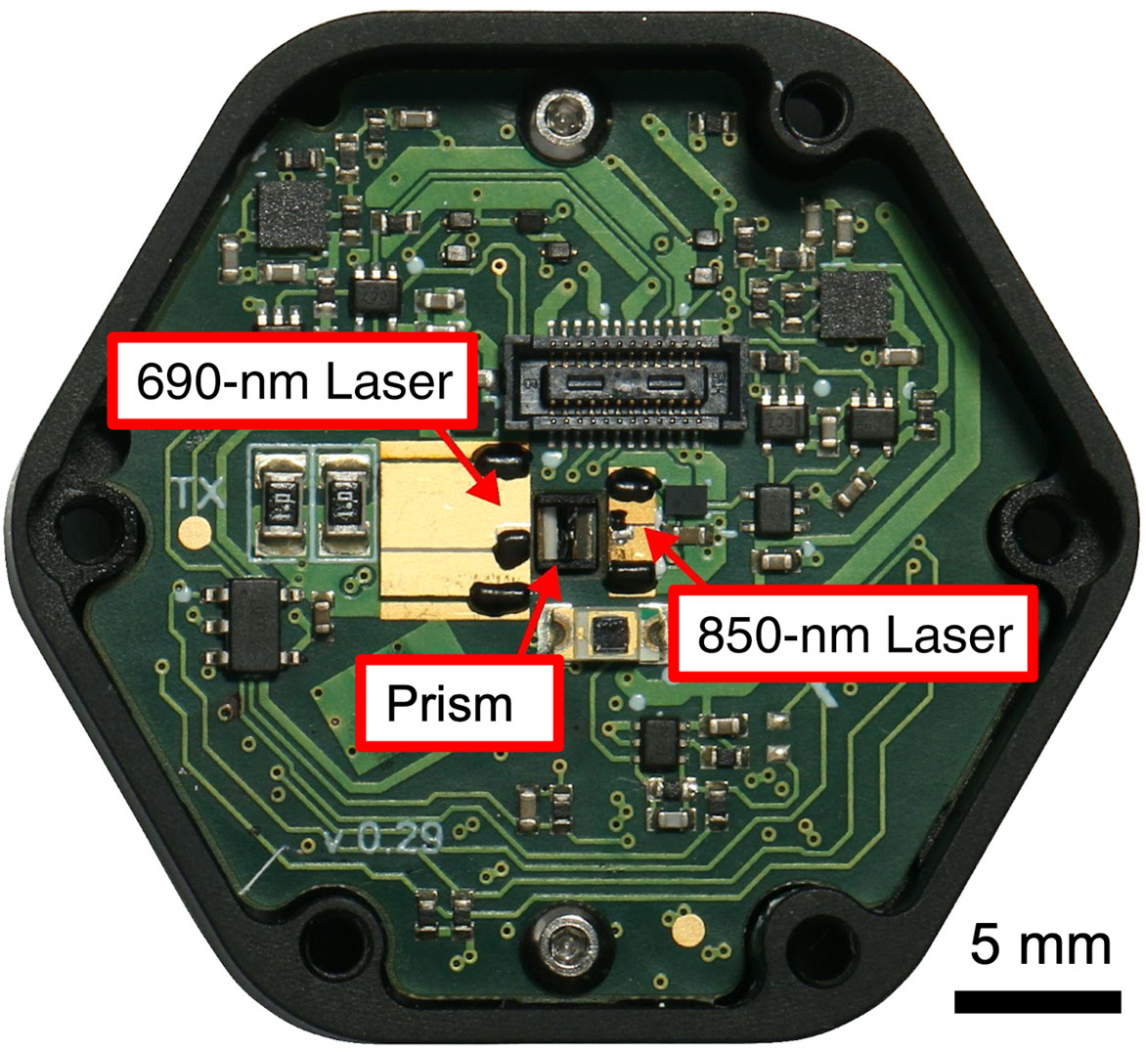
Laser subassembly showing the two different wavelengths of edge-emitting lasers, which are pulsed into a silver-coated prism to combine them into the same source light pipe. The PCB assembly is secured to an aluminum base that holds the prism in place and also serves as a heat sink for the laser diodes.

The laser module subassembly consists of a 690-nm edge-emitting laser diode and an 850-nm edge-emitting laser diode. The two laser diodes are placed at the center of the laser subassembly, where there is a cut-out in the printed circuit board (PCB; Fig. 4). This cut-out is designed to be placed over a pedestal feature of the aluminum base where the silver-coated prism is placed to redirect the edge-emitting lasers in a direction perpendicular to the PCB and into the source light guide (shown in Fig. 6). The lasers are driven by custom-designed pulse shaping circuitry that efficiently generates laser pulses that are less than 150 ps wide. The lasers operate in gain-switched mode, which enables the production of optical pulses that are shorter than the electrical pulse that drives them. The maximum average power from each laser when running at full duty cycle is limited to 5 mW. In operation, due to temporal multiplexing to avoid optical crosstalk between modules, the duty cycle for each laser is ∼4%, bringing the average power per source down to a level that classifies as a class 2 laser device according to the United States Food and Drug Administration Federal Laser Product Performance Standard Code of Federal Regulations Title 21 Section 1040.10 (US FDA FLPPS 21CFR1040.10).

In addition to the laser driver circuitry and laser diodes, this subassembly contains the power conditioning circuits for the module.

#### 2.1.3 Detector module

The detector subassembly includes six detector application-specific integrated circuits (ASICs) that are custom designed by Kernel and are optimized for performing ToF measurements for diffuse optical tomography. The key time-to-digital conversion (TDC) circuitry is integrated with the photodiodes on each of the ASICs. These custom detectors have been designed for high photon count rates, beyond $1\times 10^{9}$ counts per second, with negligible pile-up distortion. Characterization results showing this performance are discussed below.

Photon arrival times from the on-chip TDCs are accumulated into histograms and transmitted over a serial peripheral interface bus to a microcontroller. The ASICs in all modules are synchronized to a global 20-MHz reference clock, which allows for recording time-aligned signals from adjacent modules in the system. In addition, each detector ASIC has a dedicated high-voltage bias circuitry for optimally biasing each detector.

**Fig. 5:**
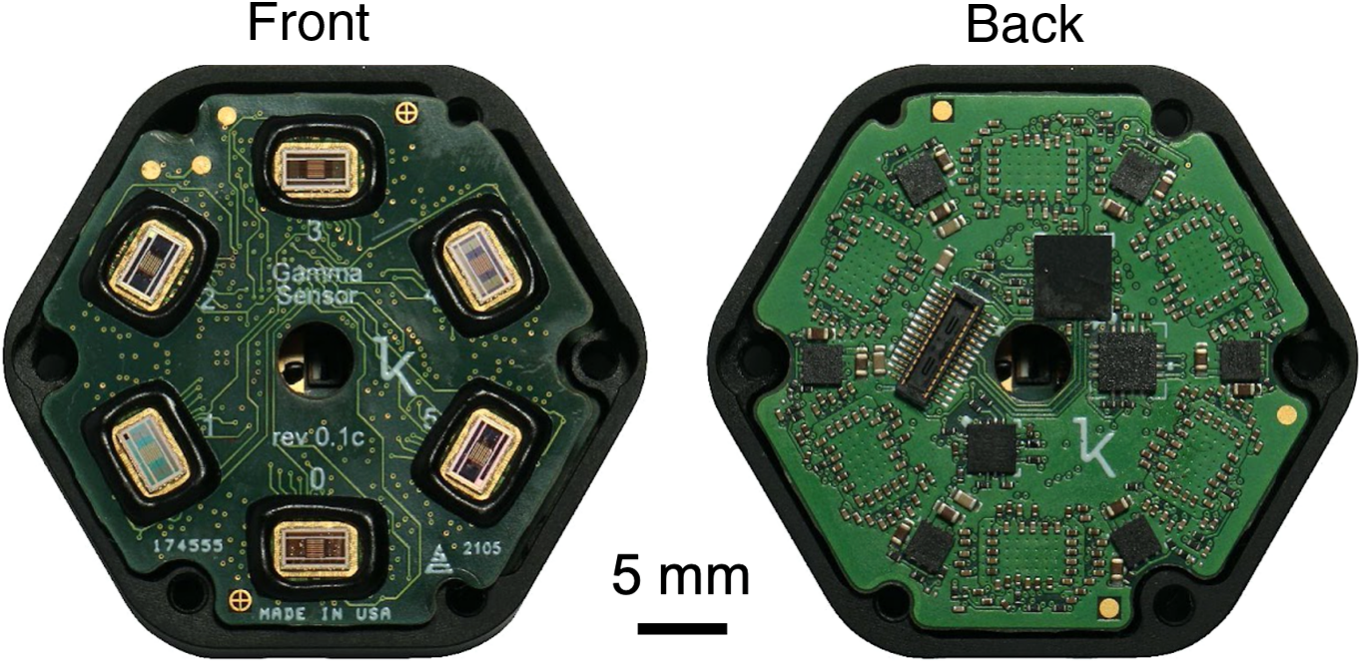
Photos of the detector subassembly showing the six Kernel custom detectors (front) and associated support circuitry (back).

One detector (labeled 0 in Fig. 5) serves as the primary detector within each module. This detector generates the trigger signals to fire the two lasers (690 and 850 nm) within each module.

The integration time for building the histogram on each detector is programmable and ranges from 1 to 800 ms per histogram. For this work, we have configured all sensors to use an integration time of 5 ms. Each histogram collected contains signal from only one wavelength. This means our histogram sampling rate is 200 Hz, and considering both wavelengths, we are able to complete spectroscopic measurements at a rate of 100 Hz. To avoid crosstalk between modules, all lasers are not enabled at the same time. This temporal multiplexing enables lasers in a 14-state pattern, completing a full cycle of data collection for all modules and wavelengths every 28 histograms, corresponding to a system sampling frequency of 7.1 Hz.

#### 2.1.4 Module optics

**Fig. 6:**
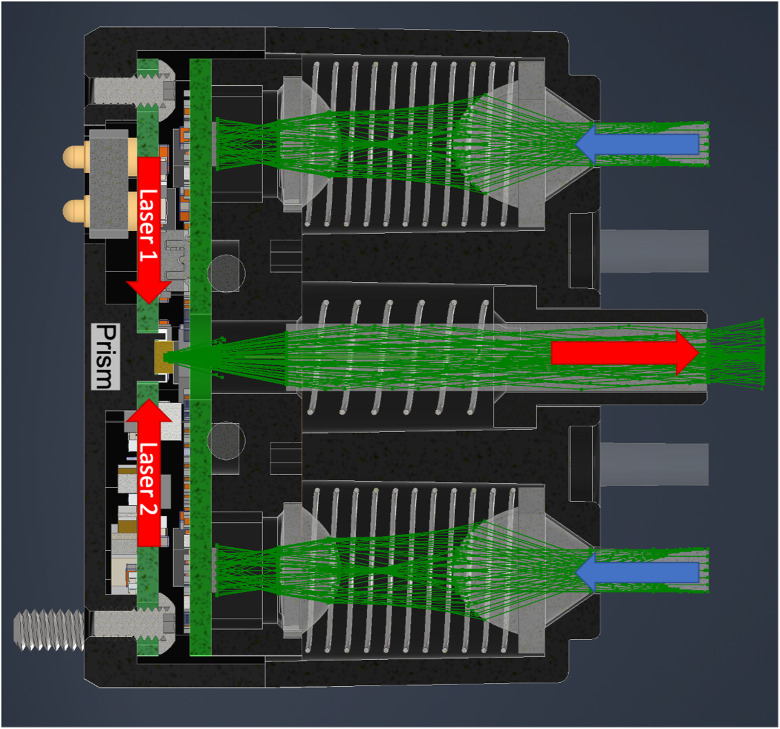
Module cross section showing the optical configuration of one of the Flow modules. The center light pipe captures the light from the two lasers (690 and 850 nm) after it is reflected off of the prism. The source light leaves the module through a 3.1-mm aperture (red arrow). The detector optics are indicated by the blue arrows and are used to image the tip of the detector light pipes onto the custom designed detectors.

The optics have been designed to serve multiple objectives—coupling the source laser light from the lasers to the scalp, capturing return light from the scalp, conforming to head curvature, mitigating interference from hair, isolating detected signal between detectors, and maintaining a stable intensity at the detector (Fig. 6).

There are a total of seven light pipes contained within the optical module with one source light pipe having a diameter of 3.1 mm and six detector light pipes each with a 2-mm diameter. The source light pipe has a numerical aperture of 0.67 and each of the detector light pipes has a numerical aperture of 0.37. Each of these seven light pipes is optically isolated from the others to prevent optical crosstalk and signal contamination.

The source light pipe (indicated by a red arrow in Fig. 6) is a single rod element that is spring loaded and hovers over the prism of the laser subassembly that is used for combining the two wavelengths of laser light into a single source spot. This light pipe is meant to be tightly coupled to the scalp of the user and light exiting from the tip of the light pipe into air will have a high divergence angle (>40 deg), which creates a safe condition should the laser inadvertently be operated when the module is not coupled to the head.

The receiving optics (indicated by the blue arrows in Fig. 6) are designed to use springs to comfortably conform to a participant’s head. The input aperture at the end of the detector light pipes is 2 mm. We have created a two-lens imaging system, consisting of one plano surface, and three aspheric surfaces, to keep the received optical intensity constant at the detector, regardless of spring compression. The 2-mm diameter light pipe that protrudes from the module also helps to comb through hair to prevent it from blocking the detectors. The light pipes can extend outside the module from 0 to 5 mm to allow for contact on curved surfaces.

### 2.2 BIP Protocol

The BIP protocol was devised to assess basic hardware performance of time-domain instruments in a standardized way.^10^ We present a subset of both protocols in which we have characterized detector responsivity, differential nonlinearity (DNL), afterpulsing, and instrument response function (IRF) along with its temporal stability.

**Fig. 7:**
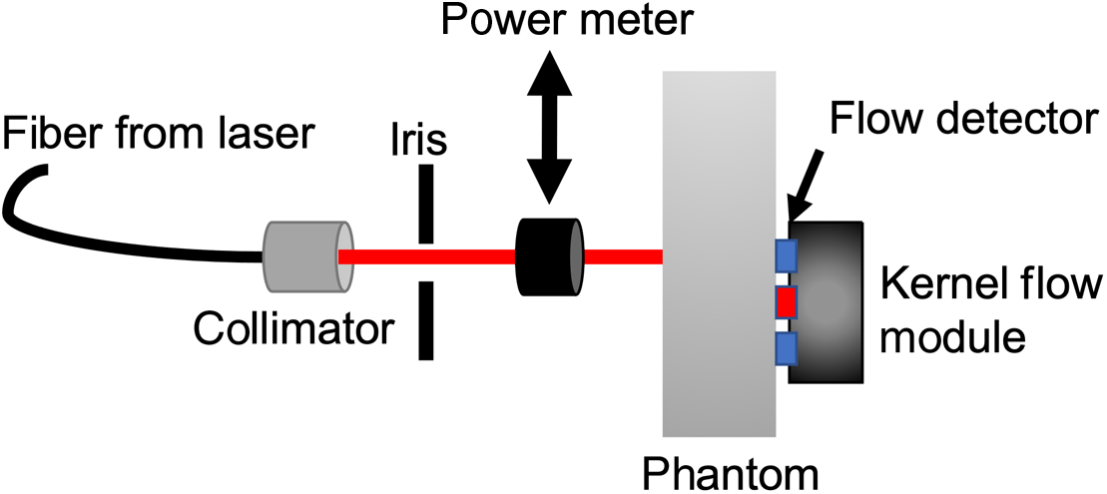
Schematic of setup used for the BIP responsivity measurement.

The detector responsivity assessment in the BIP protocol measures the efficiency of light detection for the time-domain system. The responsivity is calculated as the ratio of measured photons exiting from a calibrated phantom versus the input illumination.^10^ For a complete description of the experimental setup, we refer the reader to the original publication.^10^ Briefly, the input side of the calibrated phantom was illuminated with an external pencil laser beam with power ranging from 0.2 to 2.0 mW, and the exiting light was measured with our Kernel Flow module detector, as shown in Fig. 7.

The DNL measurement in the protocol is to characterize the nonuniformity in the time bin width of the TDCs, which results in a proportionally nonuniform number of photons attributed to each bin. The DNL was measured by applying a uniform illumination source from a battery-powered CW light source for 100 s to the detector and measuring the relative differences in the number of collected photons per bin. For our system, we found that we got the best correction factor with an illumination at $>10^{4}$ counts per bin. The deviation from the ideal equal number of photons bin to bin is calculated as the peak-to-peak difference, normalized by the mean photon counts

$$\varepsilon_{\mathrm{DNL}}=\frac{N_{\mathrm{DNL},\max}-N_{\mathrm{DNL},\min}}{\overset{¯}{N_{\mathrm{DNL}}}}.$$ (1)

**Fig. 8:**
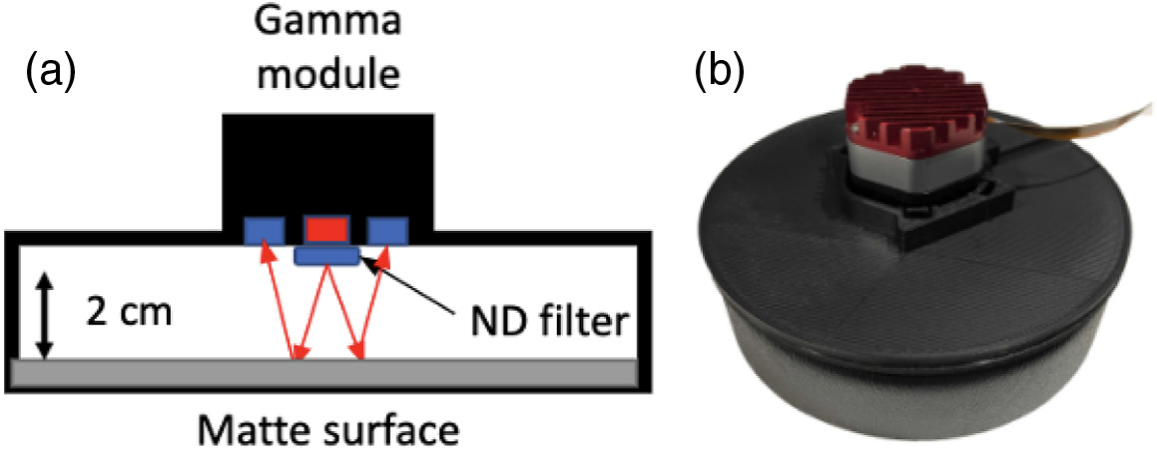
(a) Schematic of the custom fixture used to collect an IRF with the Kernel Flow module. (b) Photo of the IRF fixture.

The IRF measurement is utilized in the BIP protocol to characterize the time resolution of the Kernel Flow system. The IRF is measured with a custom fixture in reflectance mode, as shown in Fig. 8, as the geometry of the module does not allow for direct source to detector coupling. The source beam is attenuated using a neutral density filter (optical density 0.4) to avoid saturating the detectors with direct illumination, and the light is then reflected off a matte surface to redirect it into the collection optics while filling the numerical aperture. The IRF results from a convolution of the laser pulse shape and the temporal response of the detector and associated electronics. Per the protocol, the IRF was measured by averaging 20 histograms of 1-s acquisition time. Because our detector maximum integration time is only 800 ms, we collected these 20 histograms by summing 200 individual 5-ms histograms to produce a single 1-s histogram. These 20 summed histograms were then averaged according to the protocol.

From the IRF, we also calculate the afterpulsing ratio ($R_{\mathrm{AP}}$), a known signal-dependent source of noise, as defined in the BIP protocol

$$R_{\mathrm{AP}}=\frac{\left(N_{\text{mean},\mathrm{bkg}}-N_{\text{mean},\text{dark}}\right)}{N_{\text{total},\mathrm{IRF}}}\frac{T_{\text{laser}}}{\Delta t},$$ (2)

where $N_{\text{mean},\mathrm{bkg}}$ and $N_{\text{mean},\text{dark}}$ are the average counts of the background measurement in the tail of the IRF and dark count measurements taken with the module secured in the fixture of Fig. 8 and the laser off, respectively. $T_{\text{laser}}$ is the full laser period (1/repetition rate) and Δt is the time bin width. The $R_{\mathrm{AP}}$ was calculated for both 690 and 850 nm.

We also measured the stability of the IRF to determine the time scale of thermal equilibrium for a batch of 23 Kernel Flow modules in production. We recorded the stability by taking a continuous measurement starting from a cold start and recording for a period of 70 min with the Kernel Flow modules placed inside a temperature chamber at 25°C (TestEquity Model 123H). We analyze the total intensity (zeroth moment), the mean ToF (first moment), and the shape of the IRF as described in the BIP protocol.

### 2.3 nEUROPt Protocol: Depth Contrast

**Fig. 9:**
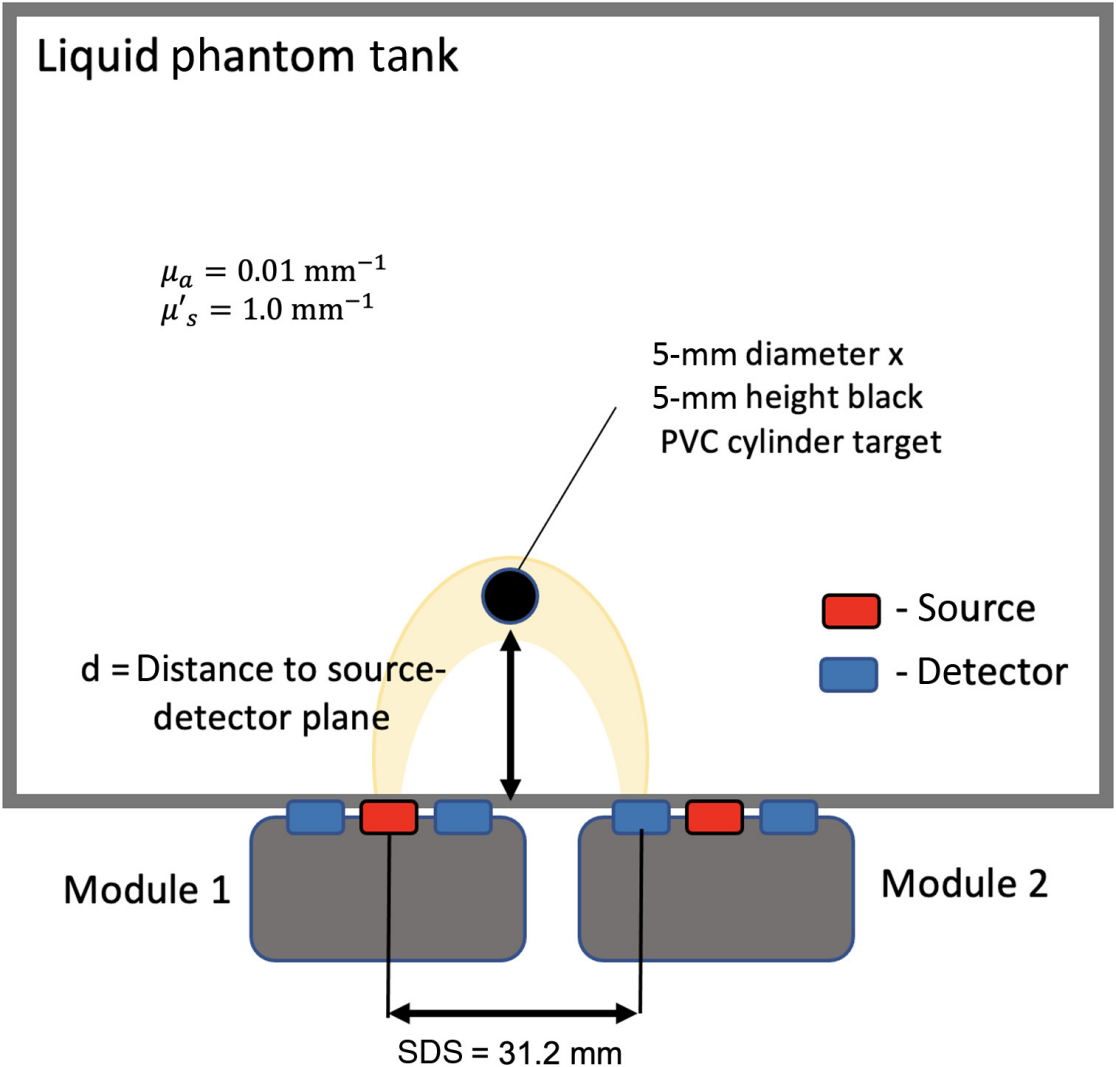
Top view schematic of the depth contrast measurement. A 5-mm diameter × 5-mm height black PVC cylinder target is measured using a single source and detector channel between two Kernel Flow modules with a 31.2-mm separation. The target is moved at different depths, at which the contrast and CNR are calculated with respect to the homogeneous liquid phantom (with $\mu_{a}=0.01\;{\mathrm{mm}}^{-1}$ and $\mu_{s}^{\prime }=1.0\;{\mathrm{mm}}^{-1}$ at 690 nm).

The nEUROPt protocol is used to assess devices at the system level using optical phantoms that mimic brain tissue.^11^ Here, we report a test from the protocol to assess the sensitivity of the Kernel Flow module using optical phantom measurements at varying distances as described in the nEUROPt protocol (those measurements aim to mimic absorption perturbations within the head). All measurements were taken for 100 s, and we used an equivalent 1-s integration time (200 consecutive 5-ms histograms) for computing contrast metrics, as in the original protocol. We use relative contrast measurements C defined as

$$C=-\frac{N_{i}-N_{o}}{N_{o}},$$ (3)

where $N_{o}$ is the reference or baseline measurement and $N_{i}$ is a measurement made after some i’th change in absorption (in our case an absorbing target) is introduced. Additionally, the contrast-to-noise ratio (CNR) is defined as

$$C=-\frac{N_{i}-N_{o}}{\sigma (N_{o})},$$ (4)

where $\sigma (N_{o})$ is the standard deviation of the reference measurement across time samples. We measured the above quantities using a black polyvinyl chloride (PVC) cylinder target of 5-mm diameter by 5-mm height placed in a liquid phantom consisting of a mixture of water, India ink, and intralipid emulsion (Sigma 20%), titrated to have background optical properties of $\mu_{a}=0.01$ and $\mu_{s}^{\prime }=1.0\;{\mathrm{mm}}^{-1}$ at 690 nm. The SDS was 31.2 mm, and the target was moved incrementally from a depth of 6 to 30 mm away from the source-detector plane. The liquid phantom tank is made of black anodized aluminum with 0.1-mm thickness mylar windows for the source and detectors, as shown in Fig. 9.

### 2.4 MEDPHOT Protocol: $\mu_{a}$ and $\mu_{s}^{\prime }$ Measurements on Optical Phantoms

**Fig. 10:**
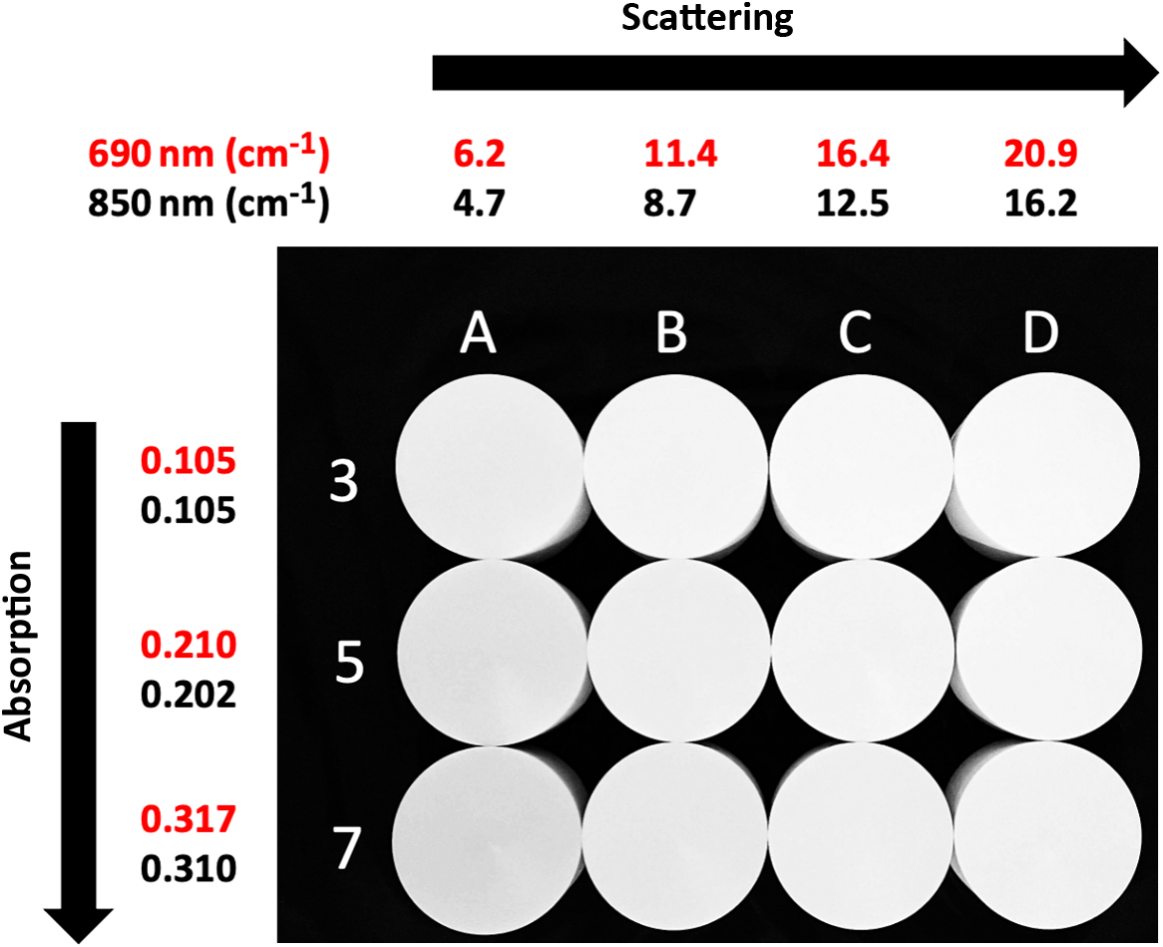
Subset of the MEDPHOT phantoms with their absorption (rows) and scattering (columns) properties. For each row/column, the average value of the absorption/scattering is shown at the two wavelengths at which the Kernel Flow system operates: 690 (red values) and 850 nm (black values) (units are ${\mathrm{cm}}^{-1}$).

The MEDPHOT protocol assesses different photon migration instruments with respect to their ability to recover known optical properties of homogeneous phantoms over a physiologically relevant range.^12^ Here, we used the Kernel Flow module to measure a subset of the MEDPHOT kit composed of 12 solid phantoms (BioPixS, Ireland). The phantom cylinders are 50 mm in height and 100 mm in diameter and consist of solid phantoms containing titanium dioxide (${\mathrm{TiO}}_{2}$) and absorbing toner in varying concentrations. The phantoms are labeled with letters (A, B, C, and D) and numbers (3, 5, and 7) in which the letters stand for a nominal scatter value and numbers correspond to absorption values, as shown in Fig. 10.

We used a single Flow module to probe the phantoms from the top in a reflectance geometry setup. We estimated the absorption and scattering by minimizing the discrepancy between the measured histogram and a predicted histogram, where the predicted histogram is the result of convolving the measured IRF with an analytical temporal point spread function (TPSF). We used the TPSF equation ^13^ derived from an analytical semi-infinite diffusion model using Robin boundary conditions. For the stability of the optical property characterization, we measured the B5 phantom in a temperature chamber at 25°C for 2 h.

### 2.5 Human Measurements

Two participants (M, 28, Fitzpatrick Type III skin, no hair; F, 38, Fitzpatrick Type II skin, medium brown hair) completed a finger-tapping task which consisted of right and left tapping blocks (duration ∼18  s) interleaved with rest blocks. Ten blocks of each hand were presented in a pseudorandomized order (e.g., Fig. 19). The study was approved by Advarra IRB (Pro00044754), and the participants gave their written informed consent before beginning the study.

Data were analyzed using custom code written in Python. Histograms from more than 2000 channels across the brain were continuously acquired during finger-tapping sessions. Channels for analysis were selected by (1) matching of the acquired histograms with shape criteria and (2) observing the heart-rate signals—a unique capability due to the high sampling rate of Flow—using both a scalp-coupling index as well as a frequency spectrum peak power measure in the heart-rate band.^14^ Histograms were used to compute the change in the absorption coefficient using the moments of the ToF distribution where the integration limits for computing the moments were set to 10% (on the rising edge) and 1% (on the falling edge) of the peak of the histogram.^15^ Subsequently, the inferred change in absorption coefficient^15^^,^^16^ was converted to the changes in the concentrations of oxyhemoglobin (HbO) and deoxyhemoglobin (HbR) signals using the modified Beer–Lambert law.^17^ Finally, HbO and HbR signals were detrended (to remove slow drifts using a moving average) and low pass filtered (finite impulse response filter, cut-off frequency=0.1  Hz) [Fig. 19(a)]. For the epoched analyses, the time window of [−5,−1]  s prior to the block onset was used for baseline correction [Figs. 19(b) and 19(c)]. To examine whether there were significant differences in the hemoglobin signals between the left- and right-tapping blocks, we used a cluster-based permutation test^18^ [Figs. 19(b) and 19(c)—middle].

## 3 Results and Discussion

### 3.1 BIP Protocol Results

**Fig. 11:**
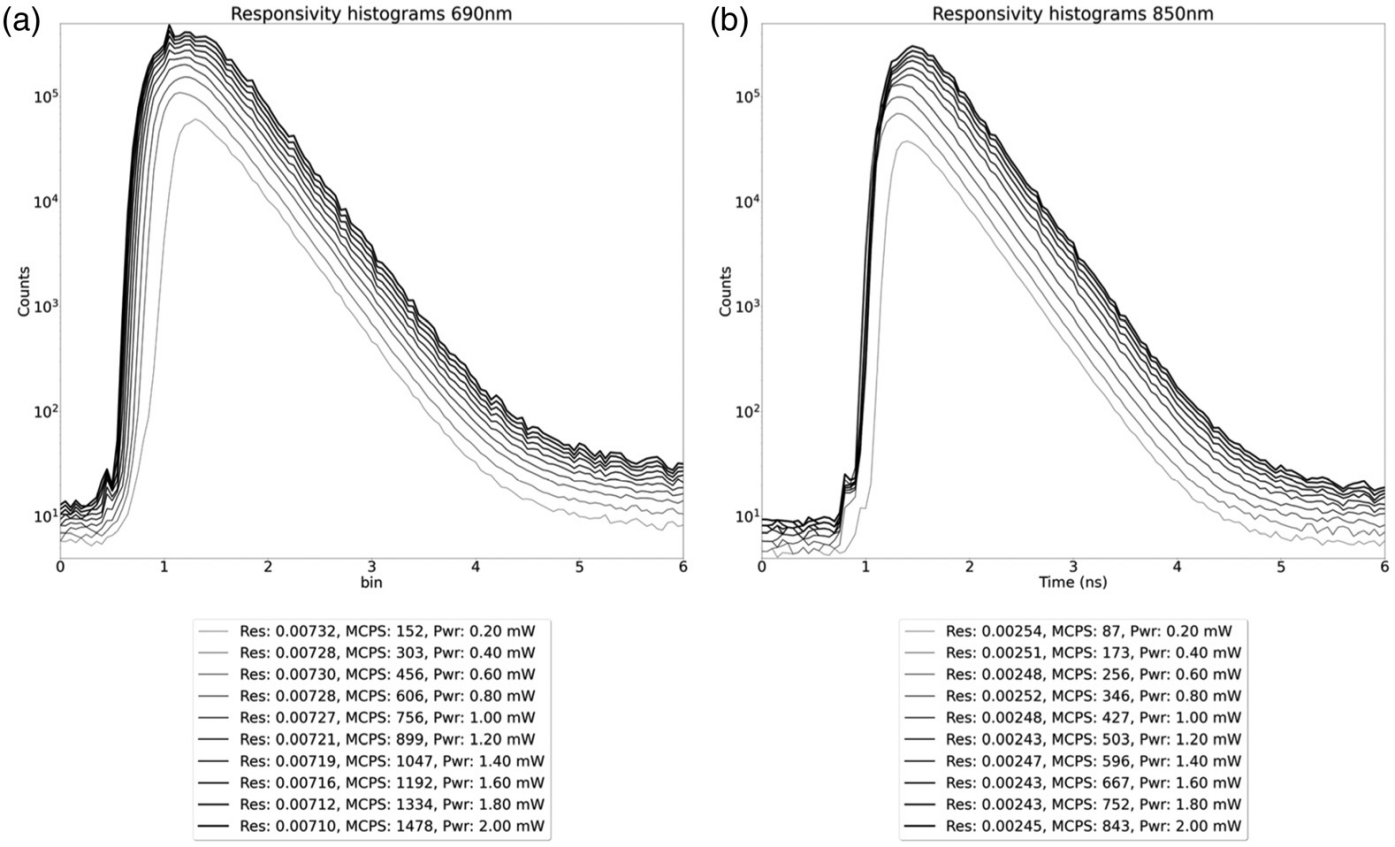
Distribution of times of flight (DTOF) of laser light transmitted through the BIP phantom for the responsivity measurement at 690 and 850 nm. Histograms with increasing counts correspond with increasing power, count rate, and responsivity.

Figure 11 shows the responsivity of the Kernel Flow detector at 690 and 850 nm with different input power ranging from 0.2 to 2.0 mW demonstrating consistent responsivity values over this range. For 690 nm, with an input of 2 mW we attain a count rate of $1.478\times 10^{9}$ counts per second. The DNL deviation in Eq. (1) was calculated as ∼0.5, so a DNL correction was applied to the histograms. At 850 nm, 2 mW of input power resulted in $0.842\times 10^{9}$ counts per second, which is expected due to silicon’s lower sensitivity at that wavelength. Throughout this section, all values are for single detectors, of which each Flow module has six.

**Fig. 12:**
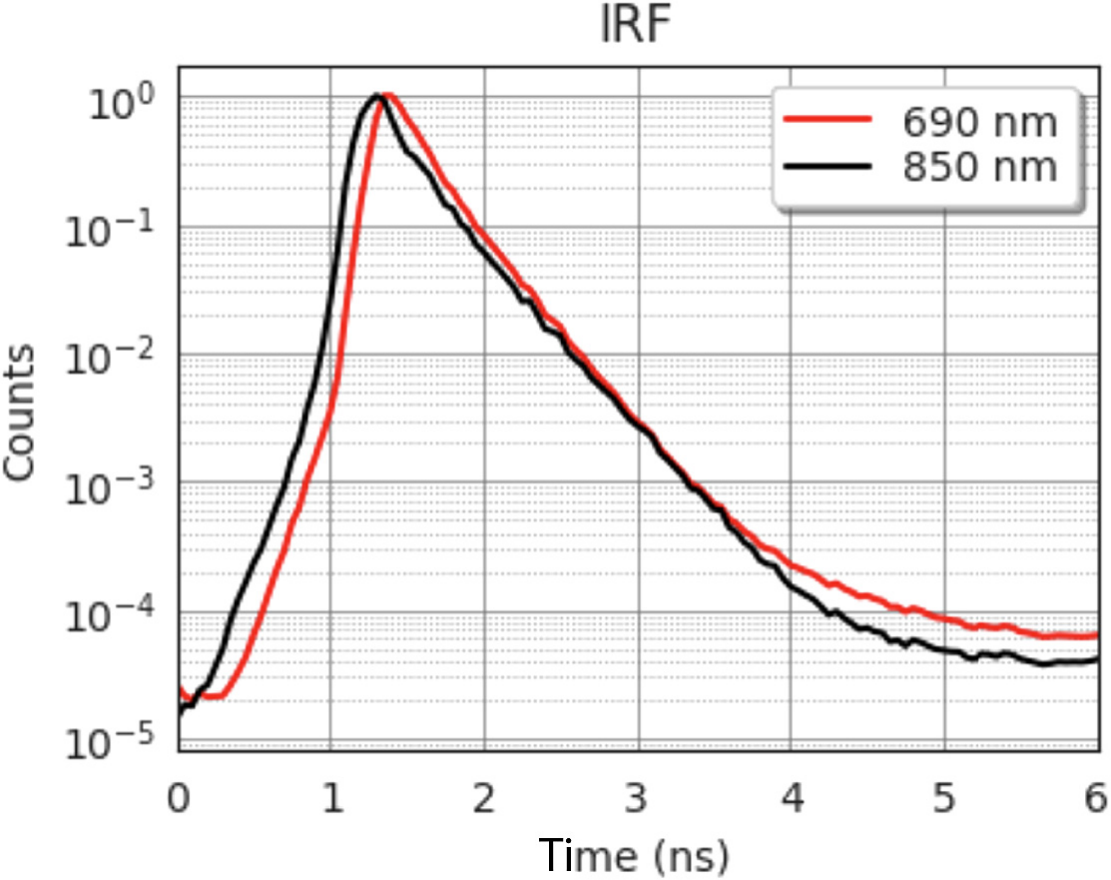
Histogram of the IRF measurement for 690 and 850 nm from a 10-mm separation source-detector pair.

Figure 12 shows the IRF collected from the Kernel Flow module. The width of the IRF at 50% [full width at half maximum (FWHM)], 10%, and 1% are 300, 770, and 1560 ps for 690 nm and 280, 790, and 1620 ps for 850 nm. This IRF was measured after a warm-up period. The $R_{\mathrm{ap}}$ was calculated to be 0.007 and 0.005 for 690 and 850 nm, respectively.

**Fig. 13:**
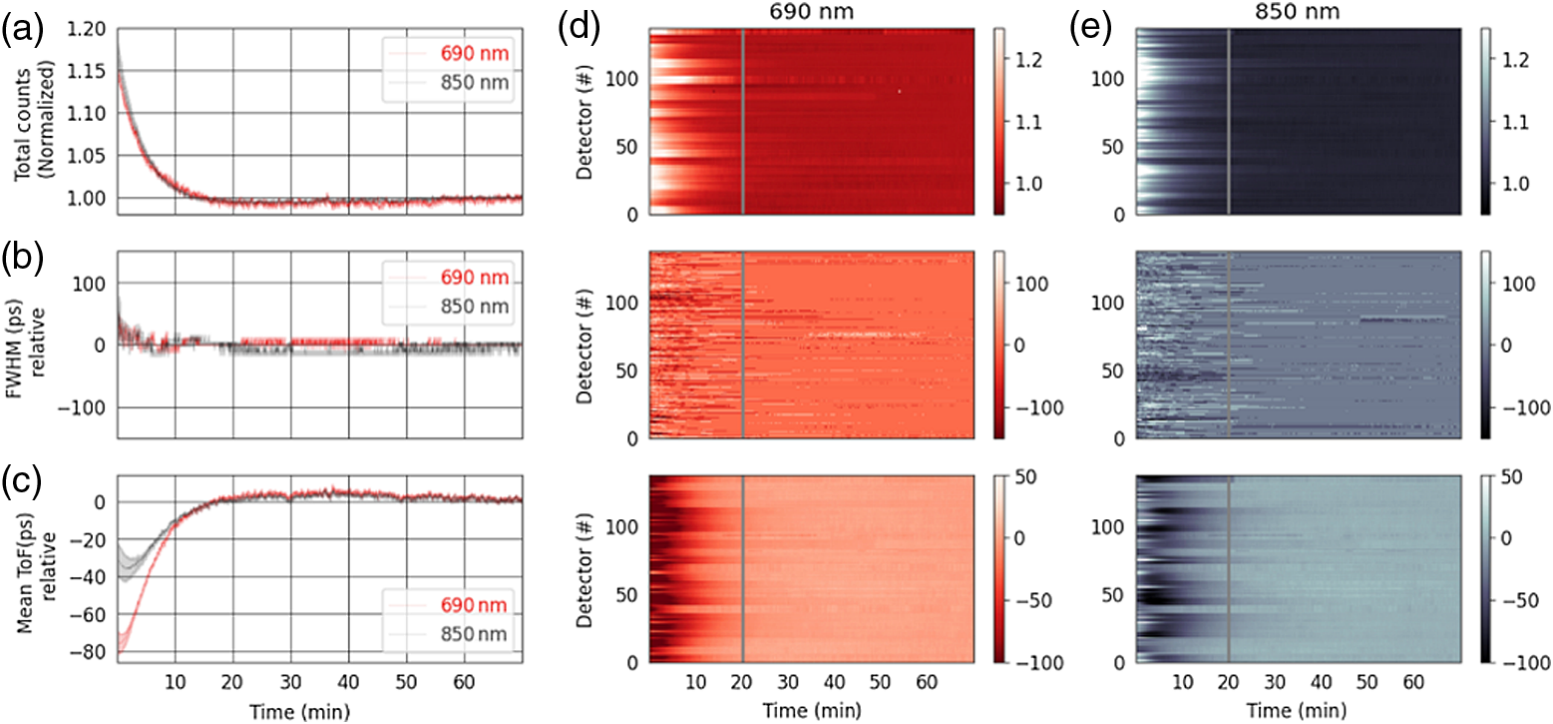
Plots of the 70-min-long IRF stability measurements showing (a) the total counts of the histogram over time for 690- (red) and 850-nm (black) measurements for a representative module (shown are mean ± standard deviation across all detectors); (b) the FWHM showing the width variation of the histograms for the module shown in (a) over time, (c) the change in the mean ToF for the module shown in (a). (d) Total counts (top), FWHM (middle), and mean ToF (bottom) for all within-module detectors for 690-nm laser (23 modules are shown). (e) Same as in (d) but for 850-nm laser.

A 70-min temporal stability measurement of the IRF is shown in Fig. 13 displaying the normalized total counts, relative FWHM change, and the relative mean ToF from 23 Kernel Flow modules affixed to a manufacturing IRF fixture. The manufacturing fixture is similar to that of Fig. 8 but with 23 modules placed inside a temperature chamber set to 25°C ambient temperature multiplexed at a frequency of 7.1 Hz (matching the sampling rate in the headset) such that there is no crosstalk between modules. The total counts for 690- and 850-nm lasers are displayed normalized to the 70-min mark and the mean ToF variability is offset to the mean value after warm up. After the warm-up period of 30 min, we see typical total counts show stability within ±2%, FWHM variability for both lasers within ±20  ps, and the mean ToF variability within ±2  ps. Modules that have poor performance are not used in the Kernel headsets.

Table 1 shows BIP protocol metrics for the Kernel Flow system and some measurements from other recent high-performance TD-fNIRS systems that have reported BIP data. These devices use different source and detector types and vary in size from being wearable to rack-mounted and bench-top systems.

**Table 1:** Comparison of Kernel Flow with other TD-fNIRS system metrics.

	Kernel Flow system			Examples from other TD-fNIRS systems
Detector responsivity	690 nm: $7.2\times 10^{-9}\;m^{2}\;\mathrm{sr}$			$1\times 10^{-9}$ to $5\times 10^{-7}$ BIP^10^
	850 nm: $2.5\times 10^{-9}\;m^{2}\;\mathrm{sr}$			$(3\text{ to }6)\times 10^{-8}$ Milan Wearable SiPM^7^
				$3.3\times 10^{-6}$ Milan Probe SiPM^19^
				$1\times 10^{-8}$ to $1\times 10^{-7}$ UCL MAESTROS^19^
				$3\times 10^{-9}$ UCL MONSTIRII^19^
DNL variation ε_DNL	<0.5			<0.04 Milan Wearable SiPM^7^
				<0.035 Milan Probe SiPM^20^
				<0.067 Milan 8-wl Rack SiPM^7^
IRF FWHM	50%	10%	1%	200 to 270 ps Milan Wearable SiPM^7^
	690 nm			308 to 556 ps Milan Probe SiPM^20^
	200 ps	770 ps	1560 ps	459 to 465 ps UCL MAESTROS^19^
	850 nm			400 to 450 ps POLIMI_2^21^
	280 ps	790 ps	1620 ps	225 ps PTB_A^3^^,^^10^
				580 ps PTB_B^3^^,^^10^
$R_{\mathrm{AP}}$	690 nm: 0.7%			687 nm: 0.21% POLIMI_2^21^
	850 nm: 0.5%			826 nm: 0.11%
IRF stability (after warm up)	N/No variability <±2%			±0.5% N/No Milan Probe SiPM^20^
	FWHM variability <±20 ps			±10 ps mean ToF
	Mean ToF variability <±2 ps			±1% N/No Milan Wearable SiPM^7^
				±2 ps mean ToF
				±1% FWHM over 15 h
				1 hr stabilize UCL MAESTROS^19^
				±5% N/No
				<±10 ps mean ToF
				>1 h to stabilize. POLIMI_2^10^
				±0.5% N/No
				±5 ps mean ToF
				<10 min stabilize. PTB_2^3^^,^^10^
				0.5% N/No.
				±10 ps mean ToF
Count rate	>1500 MCPS			40 MCPS Milan Probe SiPM^20^
				1 MCPS Milan Wearable SiPM^7^
Dynamic range	Four to five orders of magnitude (for 5 ms integration time)			Two to three orders of magnitude^7^^,^^19^^,^^20^^,^^22^^,^^23^

### 3.2 nEUROPt protocol results

**Fig. 14:**
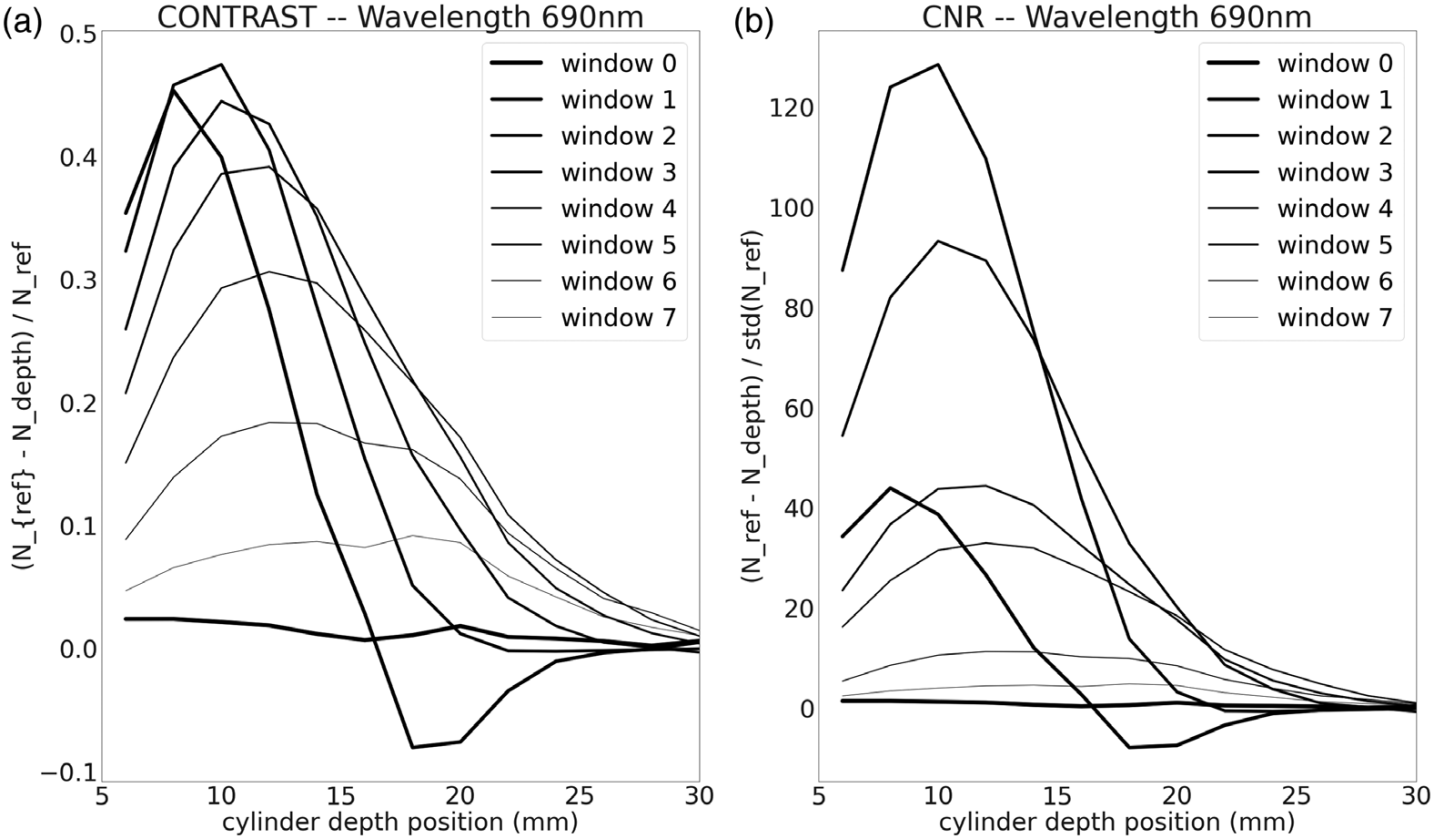
The contrast and CNR of the photon counts at 690 nm for time windows of 500 ps (starting at the rising edge of the histogram), as a 100-${\mathrm{mm}}^{3}$ volume black PVC cylinder is sequentially placed at depths from 6 to 30 mm. The histograms are from a single source-detector pair between two Kernel modules with a distance of 31.2 mm.

The contrast induced by a black $100\;{\mathrm{mm}}^{3}$ volume PVC cylinder at various depths inside a liquid phantom was calculated for a single source-detector pair formed between two synchronized Kernel Flow modules, with a SDS of 31.2 mm. Figure 14 shows the contrast and contrast to noise of 500-ps windows of the histogram as a function of depth. Photons in the early time windows are sensitive to shallower depths, while later windows have the highest sensitivity for a deeper occlusion.

### 3.3 MEDPHOT Protocol Results

#### 3.3.1 MEDPHOT: $\mu_{a}$ and $\mu_{s}^{\prime }$ measurement and fitting

**Fig. 15:**
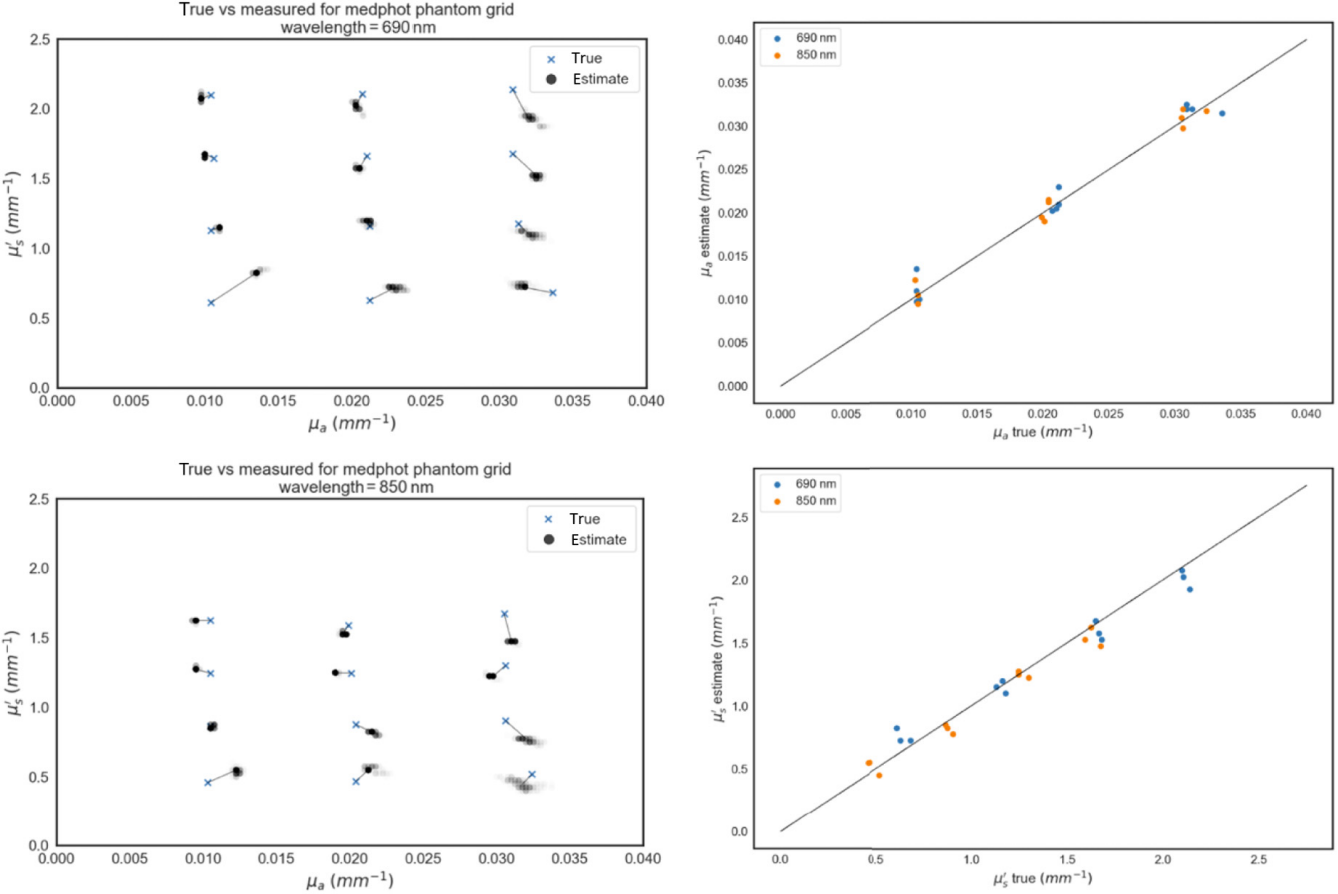
(a) and (b) The nominal $\mu_{a}$ and $\mu_{s}^{\prime }$ value of the 12 phantoms (blue x’s) and the estimated values derived from measurements by the Flow module, at 690 nm. Each 5-ms histogram generates an independent measurement, plotted in black with an opacity of α=0.01. Thus, pale gray means few histograms result in estimates at those values, whereas black indicates dozens to hundreds of estimates at that value. Thin black lines connect the nominal value to the median of estimates, to guide the eye. (a) 690-nm results and (b) 850 nm. (c) Nominal $\mu_{a}$ versus estimated $\mu_{a}$. Correlation coefficients are 0.988 and 0.993 for 690 and 850 nm, respectively. (d) Nominal $\mu_{s}^{\prime }$ versus estimated $\mu_{s}^{\prime }$. Correlation coefficients are 0.989 and 0.984 for 690 and 850 nm.

The nominal values for the 12 phantoms and their estimated values derived from Flow measurements are shown in Fig. 15. The nominal values of the phantoms are calibrated with a 2% coefficient of variation but the absolute optical properties are limited by the model uncertainties in the final manufacturer characterization. The model used in this process is based on the work described here.^24^ The measured values consist of ∼330 individual 5-ms histograms for each phantom and wavelength. We used no outlier rejection or other data cleaning. These results include every bin of every histogram from every sensor of the Flow module for each phantom measurement session.

Both the nominal optical properties and our measured values contain hard-to-quantify model uncertainties, for which there is no consensus.^20^ This uncertainty means we cannot evaluate if our measurements are accurate within phantom property uncertainties. However, as shown in Fig. 15 (right column) and Table 2, our optical property estimates are highly correlated with the nominal values, have excellent linearity, and have generally small deviations from the nominal values, averaging to 6.1% for $\mu_{a}$ and 8.2% for $\mu_{s}^{\prime }$ across wavelengths.

**Table 2:** Percent deviation of predicted values from nominal values. The nominal values correspond to the 12 phantoms.

Nominal values (${\mathrm{cm}}^{-1}$)				Deviation from nominal (%)			
690 nm		850 nm		690 nm		850 nm	
$\mu_{a}$	$\mu_{s}^{\prime }$	$\mu_{a}$	$\mu_{s}^{\prime }$	$\mu_{a}$ (%)	$\mu_{s}^{\prime }$ (%)	$\mu_{a}$ (%)	$\mu_{s}^{\prime }$ (%)
0.105	6.2	0.105	4.7	29.8	35.0	18.9	19.8
0.210	6.2	0.202	4.7	8.5	15.3	4.2	17.2
0.317	6.2	0.310	4.7	6.3	6.1	2.0	13.3
0.105	11.4	0.105	8.7	5.8	1.8	0.0	1.9
0.210	11.4	0.202	8.7	0.9	3.2	5.4	5.8
0.317	11.4	0.310	8.7	2.2	6.7	4.6	14.4
0.105	16.4	0.105	12.5	5.7	1.6	9.5	2.4
0.210	16.4	0.202	12.5	2.4	5.4	5.5	0.2
0.317	16.4	0.310	12.5	5.2	9.2	2.8	5.8
0.105	20.9	0.105	16.2	6.3	1.2	9.5	0.1
0.210	20.9	0.202	16.2	2.2	3.9	2.0	4.2
0.317	20.9	0.310	16.2	3.6	10.1	1.6	11.9
**Average deviation**				**6.6**	**8.3**	**5.5**	**8.1**

#### 3.3.2 MEDPHOT stability across time

We next examined the stability of the recovered optical properties of the phantom by recording data from a module placed on a phantom (with known optical properties) in a 2-h-long session. To calculate optical property values, we proceeded as in Sec. 2.4: We first measured the IRF in a different session (see Fig. 8 for IRF fixture), under similar temperature conditions. We then generated candidate TPSFs for different values of $\mu_{a}$ and $\mu_{s}^{\prime }$ using the analytical semi-infinite model described in Ref. 19. Convolving the measured IRF with the candidate TPSF($\mu_{a}$, $\mu_{s}^{\prime }$)’s generated candidate DTOF histograms, to be compared with the measured histograms. The $\mu_{a}$ and $\mu_{s}^{\prime }$ values that gave rise to the DTOF with the best match to the measured histogram were returned as the estimated values.

**Fig. 16:**
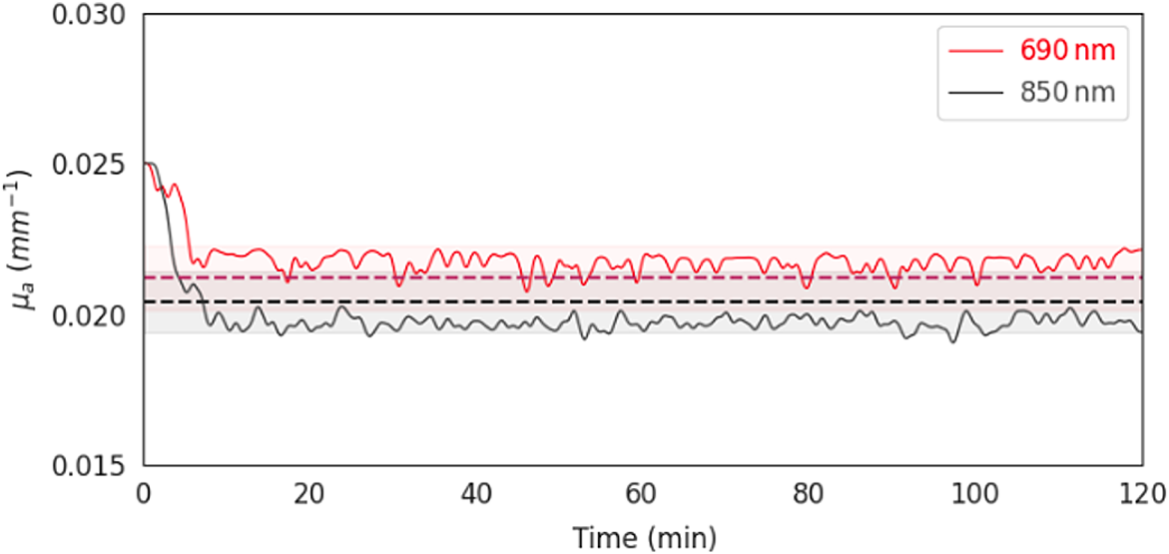
Estimated $\mu_{a}$ values of the absorption coefficient across time for the two different wavelengths using recordings from a Flow module (solid lines; red: 690 nm, gray: 850 nm). Dashed horizontal lines indicate true values and shaded areas correspond to the ±5% of the true values. Note that the recovered $\mu_{a}$ values were smoothed using a 1-min Gaussian smoothing kernel.

We show that, after a short warm-up period at the beginning, the recovered $\mu_{a}$ was stable across time and close to the true values (690 nm: $0.0218\pm 0.0006\;{\mathrm{mm}}^{-1}$, true value: $0.0212\;{\mathrm{mm}}^{-1}$; 850 nm: $0.0199\pm 0.0008\;{\mathrm{mm}}^{-1}$, true value: $0.0204\;{\mathrm{mm}}^{-1}$) (Fig. 16).

### 3.4 Human Measurements

**Fig. 17:**
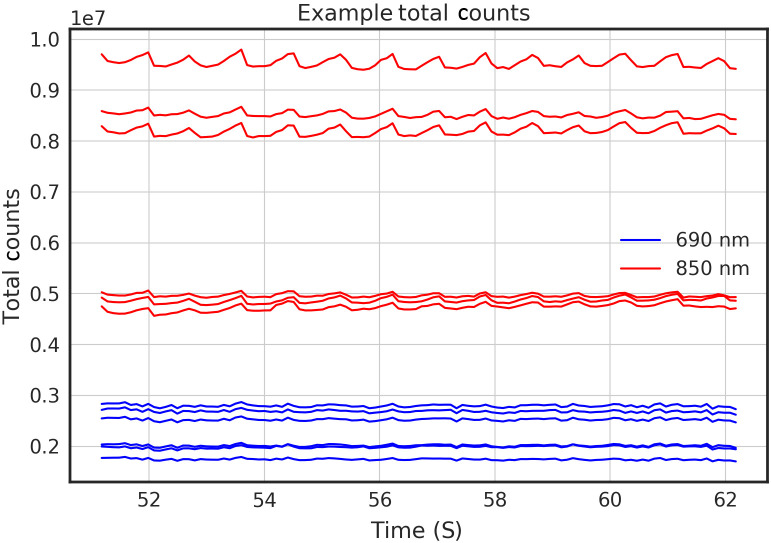
Plot showing total counts for six detectors on the forehead of a participant.

Figure 17 shows an excerpt from a recording from one module on the forehead of a participant. Traces are total counts summed over the histogram. The total counts metric shows an oscillation at a frequency consistent with a heartbeat with a higher magnitude in the 850-nm signal as compared with the 690-nm signal. The ability to measure a heartbeat oscillation with a time domain system is unique and enabled by the 200-Hz sampling rate of our detectors.

**Fig. 18:**
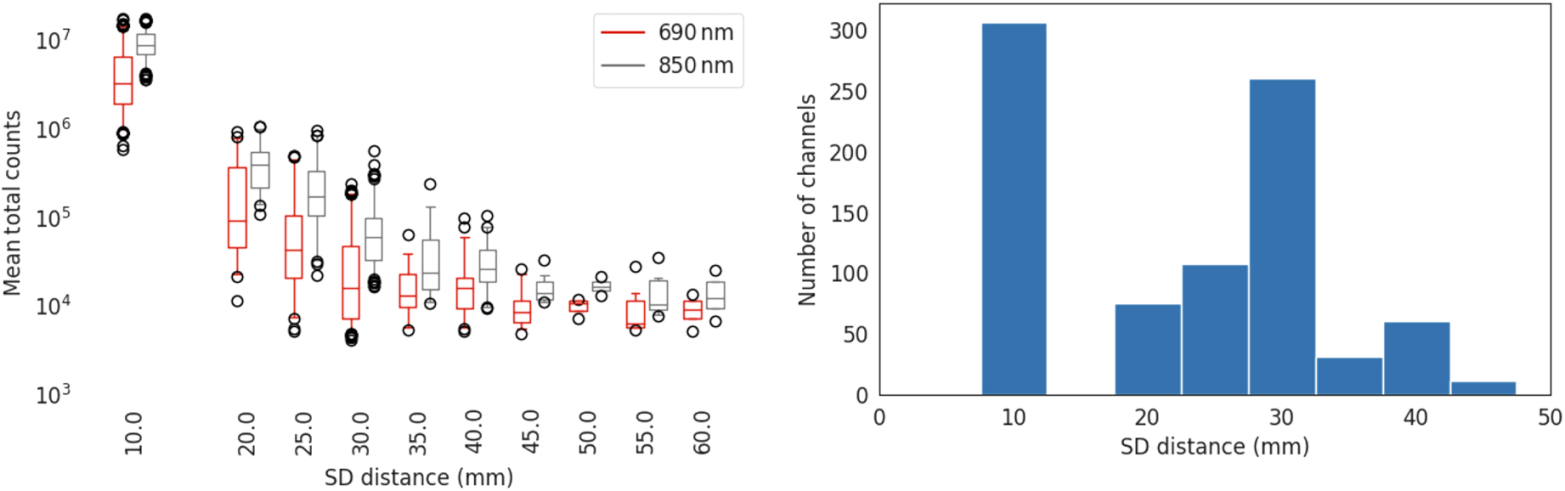
(a) The mean total counts per histogram versus the source-detector distance. (b) The number of channels that pass quality control checks at each SDS.

Channel metrics are shown for different source-detector distances in Fig. 18. In Fig. 18(a), the total counts for channels at each SDS are plotted. In Fig. 18(b), the number of channels that pass our quality control checks as outlined in Sec. 2.5 for each SDS are plotted.

**Fig. 19:**
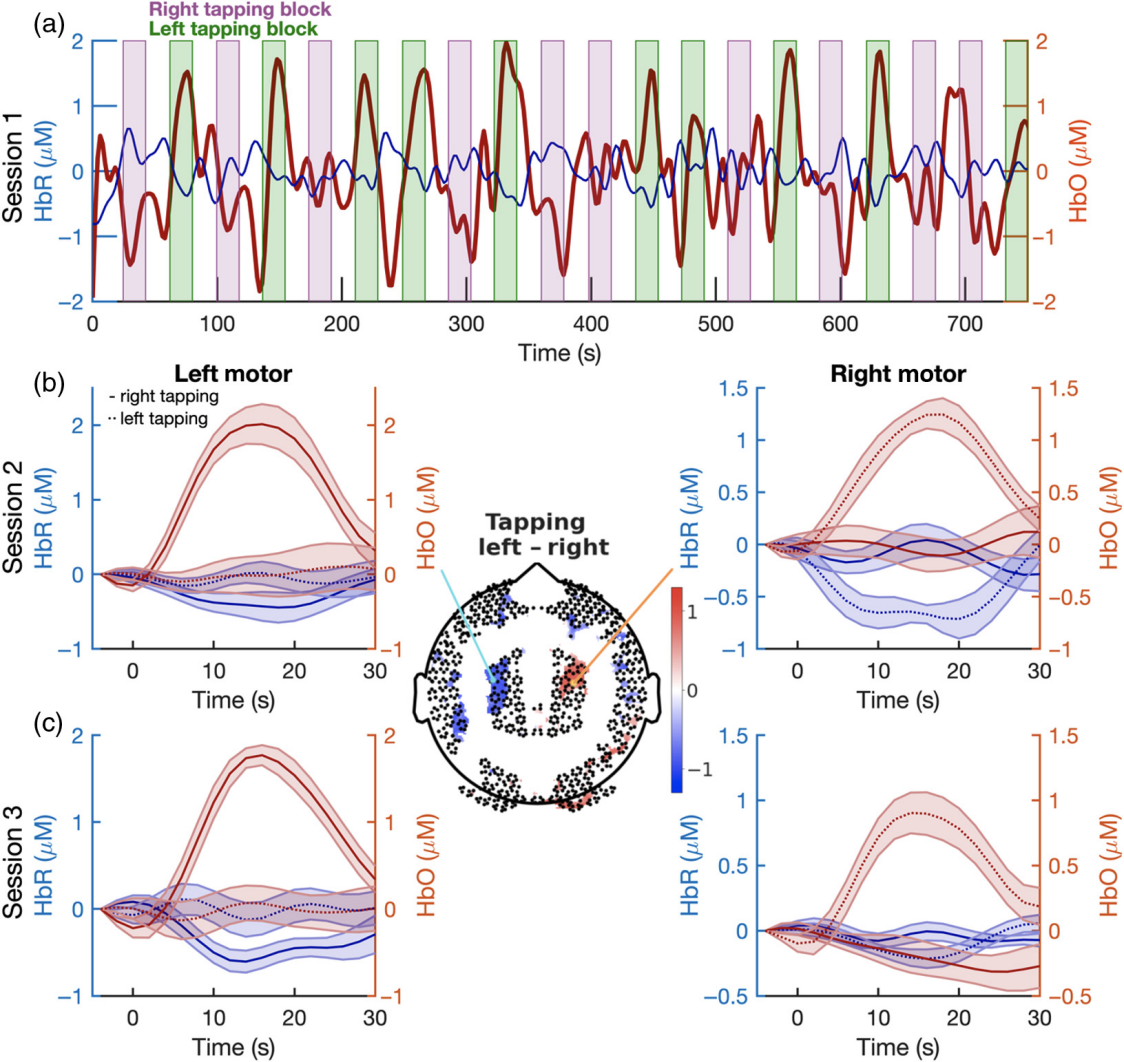
Hemodynamic responses in a finger-tapping task. (a) Time course of hemodynamic responses (HbO: red and HbR: blue) during an entire finger-tapping session from participant one. Data are from a representative channel in the right motor cortex. Note the increase in HbO signal during the left- (green) and decrease in HbO signal during the right- (purple) tapping blocks. (b) Shown are the epoched hemodynamic responses (mean ± standard error across blocks) from participant two for the example channels marked in the topographic plot (middle; light blue and orange circles correspond to the location of the channels shown in the left and right panels, respectively). Topographic plot demonstrates the results of cluster-based permutation tests that were used to assess a significant difference between the responses (HbO) during left- and right-tapping conditions. Colorbar values correspond to $-\log_{10}(p)$ multiplied by the direction of the effect. The p-values were corrected to account for multiple comparisons. (c) Same as (b) but for data from another finger-tapping session with participant two.

Hemodynamic responses for the finger tapping datasets are shown in Fig. 19 for two participants. One participant contributed one run, and the other participant contributed two runs to allow for comparison between and within participants. A whole experiment time course of a representative channel over the right motor cortex for participant one, showing HbO activation to left tapping, is in Fig. 19(a). Epoched averages of HbO and HbR in representative channels over the left and right motor cortices for participant two are shown in Figs. 19(b) and 19(c), and a topographical map showing the spatial extent of the activations is displayed in the middle of Fig. 19.

## 4 Conclusions

In this paper, we have described the Kernel Flow system and characterized it using the standard BIP and MEDPHOT protocols. We demonstrate that we have developed a wearable, whole-head coverage TD-fNIRS system that maintains or improves on the performance of existing benchtop TD-fNIRS systems. We additionally show human brain results from a commonly used validation task using Kernel Flow. Future work with Kernel Flow will involve additional human neuroscience data, including performance of the system with different hair and skin types.

## Code, Data, and Materials Availability

Data, code, and materials requests should be directed to the corresponding author at Ryan. Field@kernel.com.
